# Robustness of textural analysis features in quantitative ^99 m^Tc and ^177^Lu SPECT-CT phantom acquisitions

**DOI:** 10.1186/s40658-025-00749-0

**Published:** 2025-04-17

**Authors:** Alastair J. Gemmell, Colin M. Brown, Surajit Ray, Alexander Small

**Affiliations:** 1 College of Medical, Veterinary and Life Sciences, University of Glasgow, Glasgow, UK; 2 Department of Clinical Physics & Bioengineering, NHS Greater Glasgow & Clyde, Glasgow, UK; 3 School of Mathematics & Statistics, University of Glasgow, Glasgow, UK; 4 Department of Nuclear Medicine, Upper Ground Floor, Gartnavel General Hospital, 1053 Great Western Road, Glasgow, G12 0YN UK

**Keywords:** SPECT, Quantification, Textural analysis, Radiomics, Phantom, ^177^Lu, ^99 m^Tc

## Abstract

**Background:**

Textural Analysis features in molecular imaging require to be robust under repeat measurement and to be independent of volume for optimum use in clinical studies. Recent EANM and SNMMI guidelines for radiomics provide advice on the potential use of phantoms to identify robust features (Hatt in EJNMMI, 2022). This study applies the suggested phantoms to use in SPECT quantification for two radionuclides, ^99 m^Tc and ^177^Lu.

**Methods:**

Acquisitions were made with a uniform phantom to test volume dependency and with a customised ‘Revolver’ phantom, based on the PET phantom described in Hatt (EJNMMI, 2022) but with local adaptations for SPECT. Each phantom was filled separately with ^99 m^Tc and ^177^Lu. Sixty-seven Textural Analysis features were extracted and tested for robustness and volume dependency.

**Results:**

Features showing high volume dependency or high Coefficient of Variation (indicating poor repeatability) were removed from the list of features that may be suitable for use in clinical studies. After feature reduction, there were 39 features for ^99 m^Tc and 33 features for ^177^Lu remaining.

**Conclusion:**

The use of a uniform phantom to test volume dependency and a Revolver phantom to identify repeatable Textural Analysis features is possible for quantitative SPECT using ^99 m^Tc or ^177^Lu. Selection of such features is likely to be centre-dependent due to differences in camera performance as well as acquisition and reconstruction protocols.

**Supplementary Information:**

The online version contains supplementary material available at 10.1186/s40658-025-00749-0.

## Background

In recent years the application of Textural Analysis has seen a rise across medical imaging, where it is often termed radiomics [2]. While prominent in CT and MRI, this trend has also been seen in Positron Emission Tomography (PET) imaging, where a number of papers have focussed on its use in measuring or predicting response in lung cancer [3, 4], Neuroendocrine Tumours [5–7], and a wide variety of other tumour types [8–11]. The adoption into Single Photon Emission Computed Tomography (SPECT) has been more limited. Recently joint European Association of Nuclear Medicine (EANM) and Society of Nuclear Medicine and Molecular Imaging (SNMMI) guidelines on radiomics in Nuclear Medicine [1] have been published, covering the use of Textural Analysis in both PET-CT and SPECT-CT.

Unfortunately many clinical studies have proven not to be reproducible outside of the study centre; often the chosen Textural Analysis features or multi-factorial model will not provide the same prognostic or diagnostic benefit when applied to different patient populations or using images acquired with different parameters [12, 13]. A potential cause of this lack of reproducibility is the use of non-robust features.

Testing of quantitative accuracy in SPECT would ideally be performed with custom-built and/or anthropomorphic phantoms. However, these phantoms may require specialist 3D printing equipment or be prohibitively expensive for many centres [14]. The EANM and SNMMI guidelines on radiomics advocate the use of some simpler phantoms to test potential Textural Analysis features, recommending the use of the following:

- A uniform phantom that could consist of an empty Jaszczak phantom or similar cylindrical phantom: this can be used to test the dependency on volume of Textural Analysis features.
- A ‘Revolver’ phantom that can be constructed by binding together seven syringes and filling them with differing concentrations of a radionuclide to approximate an inhomogeneous lesion (based on the first demonstration of this phantom by Forgacs et al. [15]). When placed in a phantom with background activity, repeated scans of this Revolver insert can be used to test the repeatability of Textural Analysis features when applied to lesions.

While the guidelines are mostly focussed on application to PET-CT, there is a suggestion that the same phantom experiments could also be applied to quantitative SPECT: there has been increased interest in quantitative SPECT for clinical applications in recent years [16], with more accurate corrections for scatter, attenuation and resolution recovery helping to improve the accuracy of quantitative SPECT [17]. Potential clinical uses have been investigated, notably for ^177^Lu imaging and dosimetry in molecular radiotherapy [18–20], but also for a number of other radiopharmaceuticals [21–23]. However, many such quantitative SPECT studies have focussed on the more common quantitative parameters such as Standardised Uptake Values (SUV), with few SPECT studies directly investigating Textural Analysis. This work implements the use of phantom testing of Textural Analysis features for volume dependency and reproducibility in inhomogeneous lesions with quantitative SPECT imaging of two radiopharmaceuticals,^99 m^Tc and ^177^Lu, with the aim of identifying robust features for use in clinical studies.

## Materials and methods

### Volume dependency phantom

A uniform cylindrical phantom of height 20 cm and diameter 22 cm was filled with water (total volume = 6244 ml). The activity added was 278 MBq for ^99 m^Tc and 670 MBq for ^177^Lu, accounting for differences in photon emissions.

### Revolver phantom

**Fig. 1:**
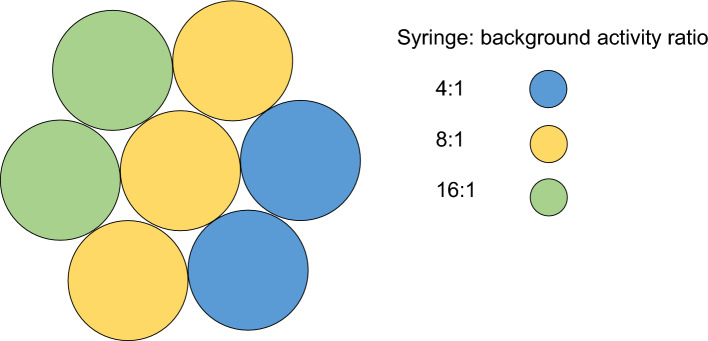
Arrangement of activity in Revolver inserts

**Fig. 2:**
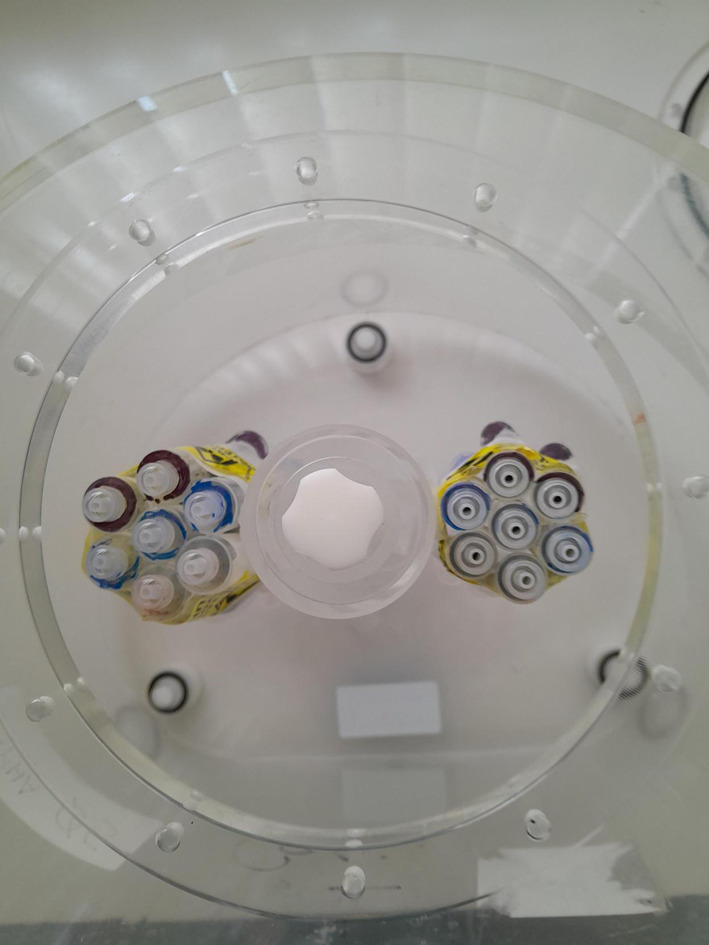
Top-down view of unfilled phantom with 2 Revolver inserts in place

The supplementary material in the EANM/SNMMI guidelines [1] describes a methodology for the use of a Revolver phantom for PET imaging with ^18^F. Given the differences in resolution between PET and SPECT imaging, it was hypothesised that the size of syringes suggested (3 ml) may be non-optimal, and partial volume effects may dominate over inhomogeneity. Therefore, for assessment of SPECT imaging multiple Revolver inserts were created, for 2.5 ml, 5 ml, and 10 ml syringes, each consisting of 7 syringes bound together in a circular arrangement. These were filled with intended syringe: background concentrations of 4:1, 8:1 and 16:1 (Fig. 1). The filled Revolver inserts were attached to the lung insert of a National Electrical Manufacturers Association (NEMA) Image Quality phantom (Data Spectrum, NC USA) [24] and placed in the body of the phantom (Fig. 2), which was then filled with background activity of 472 MBq for ^99 m^Tc and 386 MBq for ^177^Lu. For both the ^99 m^Tc and ^177^Lu filled phantoms, four consecutive scans were acquired using the acquisition parameters detailed below.

### Image acquisition

For both phantoms, images were acquired on a Siemens Intevo Bold gamma camera (Siemens Healthineers, Erlangen, Germany) using Low Energy High Resolution (LEHR) collimators for ^99 m^Tc and Medium Energy Low Penetration (MEGP) collimators for ^177^Lu. The camera had previously been calibrated for quantitative acquisitions for ^99 m^Tc and ^177^Lu using the Siemens BroadQuant software. Acquisitions used Acquire During Step with 64 views (128 projections) and 16 s/view. The energy window for ^99 m^Tc was 140 keV ± 15% with a lower scatter window width of 15% immediately adjacent to the primary window. The energy windows for ^177^Lu were 113 keV ± 15% (with a lower scatter window width of 15% immediately adjacent to the 113 keV window), and a second primary window of 208 keV ± 15%. Quantitative reconstruction used the Ordered Subset Conjugate Gradient method with parameters that had been optimised locally for quantitative accuracy (for ^99 m^Tc – 48 iterations, 1 subset and 5 mm filtering; and for ^177^Lu-72 iterations, 1 subset and 0 mm filtering).

### Feature extraction

Image analysis was performed using Lifex v6.3 [25] (www.lifexsoft.org): pixel size was 1.95 × 1.95 × 1.95 mm with discretisation to 64 bins (as recommended in [10] and [26]) and SUV range of 0–20 to give bin width of 0.3 SUV/bin (as per [26]). The choice of a fixed bin width and ~ 2 mm isotropic voxel size meets the recommendations of Hatt et al. [27], and also showed the most reproducible features in one study of PET Textural Analysis [28].

For the uniform phantom, 29 spherical Volumes of Interest (VOIs) of increasing size from 0.9 ml up to 1021 ml (diameters from 10 to 122 mm, in 4 mm increments) were placed expanding from the centre of the phantom and avoiding the phantom edges. Sixty-seven Textural Analysis features compliant with the IBSI [29] (and applicable to SPECT imaging), were extracted. The measured values were correlated against VOI volume graphically for visual interpretation.

For the Revolver phantom, VOIs using a threshold SUV of 2.5 times the background was drawn separately over each Revolver insert, from which the 67 features were extracted. The Coefficient of Variation (CoV) was measured across the four scans for each feature (and each Revolver size and radionuclide) then compared to the suggested upper threshold of 10% from the supplementary material of the EANM/SNMMI radiomics guidelines [1].

### Feature reduction

It is important that any feature selected for clinical analysis should behave similarly across a range of volumes, as tumour size will vary considerably between patients and diseases. The graphs of volume dependency for each feature (supplementary material) were visually inspected to determine those features that showed non-converging behaviour (as described by Forgacs et al. [15]) and hence should be excluded from further analysis. Those features with a known or intrinsic correlation with volume (e.g. SUVs and shape-related features such as sphericity) but have been shown to have clinical value [30, 31] and are in common usage within the literature were not excluded.

Features must also be robust to repeat measurements (for example to allow confidence in assessing changes following treatment), therefore for the Revolver phantom experiment, those features that showed CoV > 10% in any Revolver insert were excluded.

## Results

### Volume dependency

**Fig. 3:**
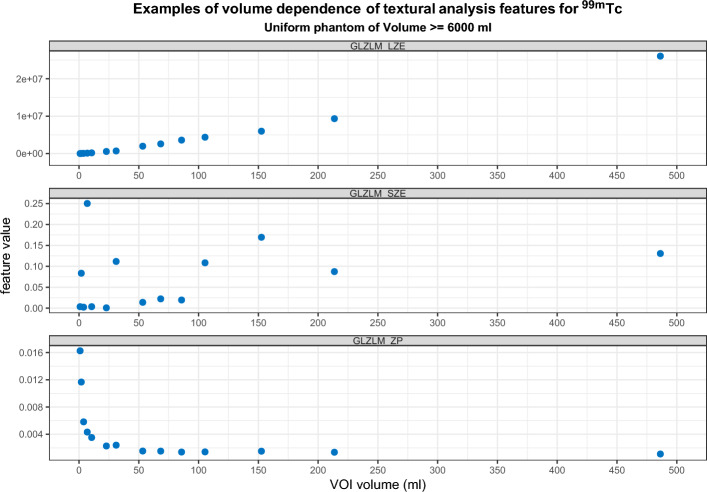
Examples of (top) positive correlation; (middle) random distribution; and (bottom) convergence with increasing volume

Graphs displaying the volume dependency against 67 features for both radionuclides (up to a VOI of 150 ml) are contained in the supplementary information. Forgacs et al. demonstrated three typical patterns of volume dependency [15]: Fig. 3 shows examples of these three patterns taken from this work. All converging features showed a volume dependency at volumes below approximately 25 ml (Table 1). Features that showed convergence with increasing volume were identified by visual inspection (supplementary material).

**Table 1:** Non-converging features following volume dependency testing

^99 m^Tc	^177^Lu
SUVQ1	SUV Skewness
SUVQ2	SUV Kurtosis
SUVQ3	SUV Excess Kurtosis
Discretised SUVmean	Discretised SUVmin
Discretised SUVmax	Discretised SUVmax
Discretised TLSRE	Discretised SUVQ3
GLCM homogeneity	Discretised Skewness
GLCM contrast	Discretised SUVpeak 0.5 ml
GLCM dissimilarity	Discretised SUVpeak 1 ml
GLRLM LGRE	Discretised TLSRE
GLRLM HGRE	GLRLM HGRE
GLRLM SRLGE	GLRLM GLNU
GLRLM GLNU	GLRLM RLNU
GLRLM RLNU	NGLDM Busyness
NGLDM Contrast	GLZLM SZE
NGLDM Busyness	GLZLM LZE
GLZLM SZE	GLZLM LGZE
GLZLM LZE	GLZLM HGZE
GLZLM LGZE	GLZLM SZLGE
GLZLM HGZE	GLZLM SZHGE
GLZLM SZLGE	GLZLM LZLGE
GLZLM SZHGE	GLZLM LZHGE
GLZLM LZLGE	GLZLM GLNU
GLZLM LZHGE	GLZLM ZLNU
GLZLM GLNU	GLRLM LRE
GLZLM ZLNU	

### Reproducibility in inhomogeneous volume

**Fig. 4:**
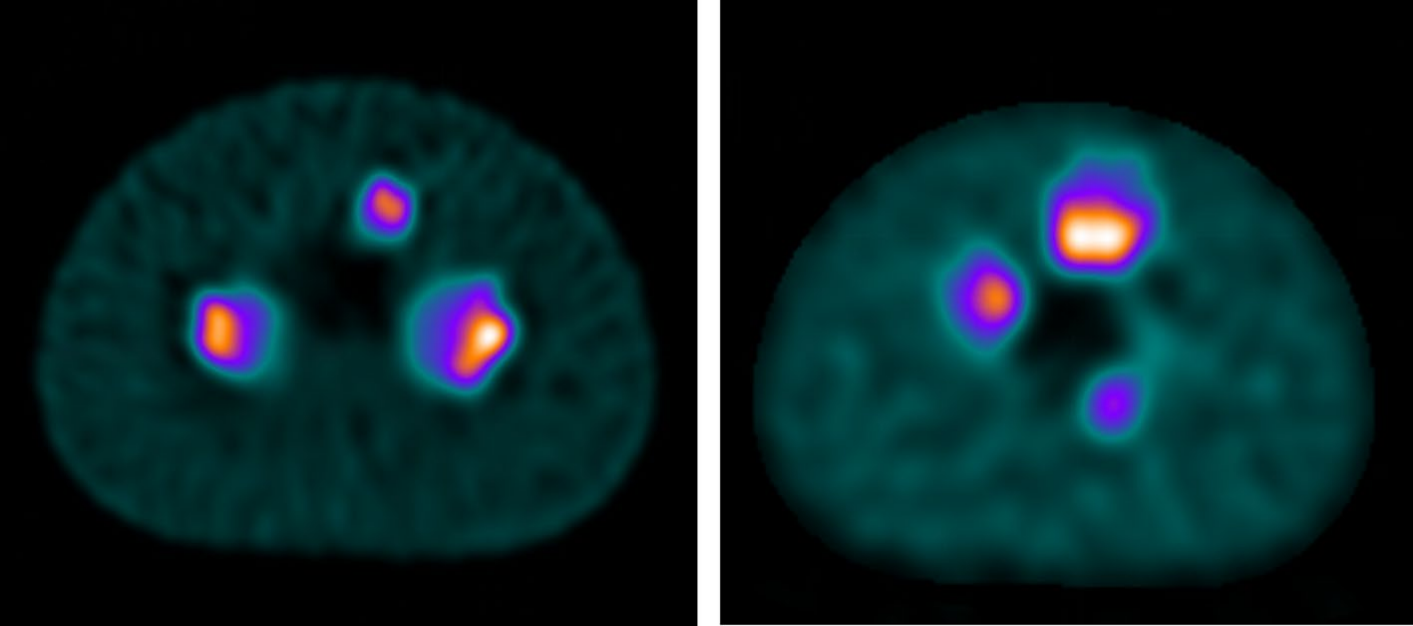
Axial slices of (left) ^99 m^Tc and (right) ^177^Lu Revolver phantoms

An example slice from the reconstructed images is shown in Fig. 4 for each of the radionuclides tested. The average volume of the VOI after application of thresholding is shown in Table 2.

**Table 2:** Average VOI size after application of 40% SUVmax threshold: mean ± standard deviation

Revolver insert (syringe size)	^99 m^Tc	^177^Lu
2.5 ml × 7	33.3 ± 0.5 ml	30.3 ± 1.3 ml
5 ml × 7	66.3 ± 1.0 ml	64.0 ± 3.7 ml
10 ml × 7	141.0 ± 1.1 ml	121.9 ± 6.7 ml

The features that exceed the 10% CoV threshold suggested by the EANM/SNMMI radiomics guidelines are summarised in Tables 3 and 4 for each Revolver insert, where multiple failures across the three Revolver sizes are emphasised.

**Table 3:** Textural analysis features with coefficient of variation > 10% for ^99 m^Tc

CoV > 10% in 1 Revolver insert	CoV > 10% in 2 Revolver insert2	CoV > 10% in 3 Revolver inserts
Discretised SUV excess Kurtosis	Discretised Histogram Excess Kurtosis	
GLZLM LZE	SUV excess Kurtosis	
GLZLM LZLGE		
GLZLM SZHGE		
GLZLM SZLGE		
GLZLM ZLNU		
NGLDM Busyness

**Table 4:** Textural analysis features with coefficient of variation > 10% for ^177^Lu

CoV > 10% in 1 Revolver insert	CoV > 10% in 2 Revolver insert2	CoV > 10% in 3 Revolver inserts
Compacity	Discretised Histogram Kurtosis	Discretised histogram Excess Kurtosis
Discretised SUV Kurtosis	GLZLM SZE	Discretised SUV excess Kurtosis
Discretised SUV Skewness	GLZLM SZHGE	GLCM Energy
Discretised SUVmin	GLZLM ZP	GLZLM GLNU
GLZCM contrast	NGLDM Busyness	GLZLM HGZE
GLRLM LGRE	NGLDM Contrast	GLZLM LGZE
GLRLM LRLGE	SUVmin	GLZLM LZE
GLRLM SRLGE		GLZLM LZLGE
SUV Kurtosis		GLZLM SZHGE
SUV Skewness		GLZLM SZLGE
		GLZLM ZLNU
		SUV Excess Kurtosis

### Final feature reduction

The remaining features from the volume dependency and Revolver phantoms were combined to produce a list of remaining features: these features were suggestive for future exploration in clinical studies using data acquired and reconstructed with the local parameters as listed in Table 5: it consists of 39 features for ^99 m^Tc and 33 features for ^177^Lu.

**Table 5:** Remaining features following reduction

^99 m^Tc	^177^Lu
SUVmin	SUVmin
SUVmean	SUVmean
SUVstd	SUVstd
SUVmax	SUVmax
SUV Skewness	SUVQ1
SUV Kurtosis	SUVQ2
SUVpeak 0.5 ml	SUVQ3
SUVpeak 1.0 ml	SUVpeak 0.5 ml
TLSRE	SUVpeak 1.0 ml
Discretised SUVmin	TLSRE
Discretised SUVstd	Discretised SUVmean
Discretised SUVQ1	Discretised SUVstd
Discretised SUVQ2	Discretised SUVQ1
Discretised SUVQ3	Discretised SUVQ2
Discretised SUV skewness	Discretised histogram skewness
Discretised SUV kurtosis	Discretised histogram entropy log10
Discretised SUVpeak 0.5 ml	Discretised histogram entropy log2
Discretised SUVpeak 1.0 ml	Discretised histogram energy
Discretised histogram energy	Sphericity
Discretised histogram skewness	Surface area
Discretised histogram kurtosis	GLCM homogeneity
Discretised histogram entropy log10	GLCM correlation
Discretised histogram entropy log2	GLCM entropy log10
Sphericity	GLCM entropy log2
Surface area	GLCM dissimilarity
Compacity	GLRLM SRE
GLCM energy	GLRLM SRHGE
GLCM correlation	GLRLM LRHGE
GLCM entropy log10	GLRLM RP
GLCM entropy log2	NGLDM coarseness
GLRLM SRE	NGLDM contrast
GLRLM LRE	GLZLM ZP
GLRLM SRHGE	Volume
GLRLM LRLGE	
GLRLM LRHGE	
GLRLM RP	
NGLDM coarseness	
GLZLM ZP	
Volume	

## Discussion

With the recent publication of EANM guidelines for quantitative SPECT-CT [32], there is greater emphasis on the harmonisation of quantitative SPECT-CT; though further work is needed to harmonise acquisition and reconstruction parameters in the same way that EARL accreditation aids harmonisation for PET-CT [33]. This paper confirms that the methodology outlined in EANM and SNMMI guidelines on radiomics [1] are applicable to SPECT, though the specific features selected should be confirmed by testing at other centres, due to differences in acquisition and processing parameters as well as inherent differences in camera performance that may affect reproducibility (as demonstrated in PET radiomics [34]).

For some features, a direct dependence on volume is to be expected (e.g. for surface area) although that in and of itself is not a reason for exclusion from further analysis, as it may be of clinical value. For example, two tumours may have the same volume, but an irregular-shaped lesion would have a greater surface area. This may have clinical implications: one study has proposed that the density of somatostatin receptors would be higher for such an irregular-shaped tumour, therefore somatostatin receptor radiopharmaceuticals would show greater uptake, and hence a feature such as low sphericity could be predictive [31]. Some calculated features (such as those that combine SUV and volume, for example, Total Lesion Somatostatin Receptor Expression) are also specifically constructed so as to be dependent on volume and have been shown to have predictive value in patients [35].

As well as theoretical limitations of testing and excluding features due to volume dependence (as outlined in the point above), the practical implementation of testing with spherical VOIs in an image made up of rectangular voxels also has drawbacks. The VOI will not be truly spherical, hence affecting the measurement of some features, e.g. sphericity. The pixelation will be more prominent at lower volumes and may imply a volume dependence not truly inherent to the feature. Spherical lesions are also unlikely to be delineated in clinical studies.

A study on tumoral homogeneity [36] used a mathematical framework to calculate a minimum tumour size for accurate inhomogeneity measurement in PET of 45 ml (though dependent on their assumptions in imaging parameters). They propose that this limit is not just influenced by technical factors, but also the biology of the tumour, hypothesising that smaller tumours will not have sufficient size to allow for the potential biological variations (e.g. hypoxia, necrosis) that result in inhomogeneity to exist [36]. The limitations of their minimum volume calculation, as highlighted by Hatt et al. [27] are that it was performed only for a single feature (GLCM entropy), fixed bin size quantisation was used and testing was only in 2D. It has been shown that using fixed bin width (used in this study) instead of fixed bin size is that it can reduce the correlation of features with volume [37]. A different study looking at both simulated spherical lesions and multiple cohorts of patient images found (for a limited set of features) that while a volume dependency did exist, the correlation was limited above volumes of 10 ml, and useful information could be extracted, potentially down to volumes greater than 3 ml – for example with Entropy, Dissimilarity, or Zone Percentage [10]. The work reported here did show similar results for these features, with reducing correlation with increasing volume for ^177^Lu however, the results were not replicated for ^99 m^Tc. The work here was done with spherical VOIs in a uniform phantom however and may not be directly comparable to segmented lesions in patients, as in the original study.

The importance of the number of voxels, rather than volume, is emphasised by Orlhac et al. in their guide to implementing Textural Analysis in PET [26]; they suggest a minimum of 64 voxels is required in a VOI in order to calculate robust features (for the pixel size in this study that would correspond to a minimum volume size of 0.5 ml – smaller than the VOIs used in this study).

**Fig. 5:**
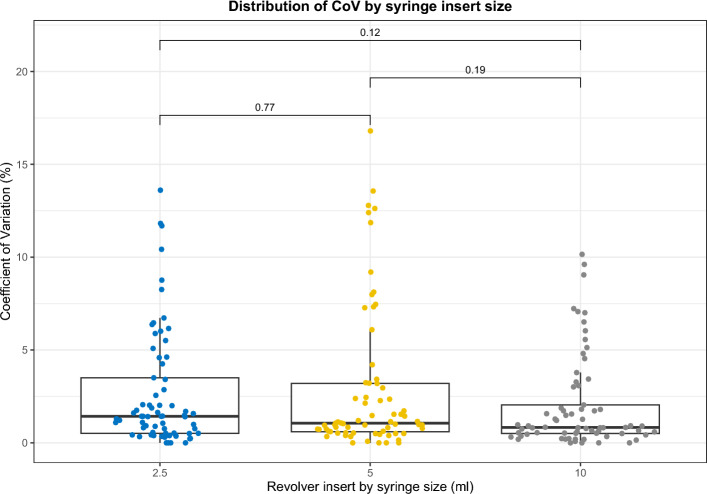
Distribution of CoV results for ^99 m^Tc by syringe size

An increasing size of the Revolver insert did not appear to decrease variability in the feature values: more features exceeded the CoV threshold of 10% for the 5 ml syringes than the 2.5 ml, for both radionuclides. There was no significant difference between the distributions of CoV results for any of the three Revolver insert sizes (Fig. 5). The higher number of features exceeding CoV > 10% for ^177^Lu compared to ^99 m^Tc may be due to higher noise in the images, potentially caused by the lower frequency of gamma emissions from ^177^Lu than ^99 m^Tc, or due the differing number of iterations and post-reconstruction filtering applied in the optimised reconstructions for each radionuclide. However, results from uniform phantom acquisitions where both radionuclides were reconstructed with the same reconstruction parameters showed that ^177^Lu had a higher CoV than ^99 m^Tc (20.2% versus 14.3%, p < 0.001) and highlights the higher noise in ^177^Lu images.

This study does not investigate the impact of segmentation on reproducibility of features: neither the impact of differing segmentation algorithms nor inter-operator variability. Experimental evaluation of the effect of different segmentation algorithms would be a significant undertaking, given the extensive variety of methods available – see Foster et al. for a review of segmentation algorithms applied to PET [38]. This study utilises an adaptive threshold based on 2.5 times the background intensity, as prescribed in the Revolver phantom instructions in the EANM/SNMMI guidelines’ supplementary information [1]. The advantages of this method are the simplicity of application using a variety of processing platforms, and that it is adaptable to changes in local background: an important factor in clinical studies. An issue for adaptive threshold segmentation can be the exclusion of necrotic areas of tumours, which may show reduced uptake centrally [39]. Inter-operator variability for segmentation is likely to be small for a SPECT phantom study using an adaptive threshold method; in clinical SPECT studies the variability in a segmented volume may be higher, due to difficulties in agreeing on the tumour geometry [40]. There is also likely to be inter-feature correlation (particularly between discretised and non-discretised versions of the same feature), however this is better tested in analysis of clinical images, where other highly correlated features can also be identified [4].

Phantom acquisitions may not provide a sufficient representation of the volumetric complexity and heterogeneity of lesions in the clinical setting – spherical inserts or, in this particular study, syringes do little to mimic the appearance of clinically relevant lesions. The use of 3D-printed lesions, designed from imaging of actual tumours would help phantom studies more closely replicate clinical imaging [41]. Factors such as patient movement, respiratory motion and physiological excretion are also not incorporated into most currently available phantoms. The identification of robust radiomic features by phantom study is just one step in the path to clinical translation, with significant issues identified in several review articles and editorials [42, 43].

It is clear from Tables 3 and 4 that certain ‘families’ of feature were particularly prone to higher CoV and could be chosen to be excluded from further analyses (notably Excess Kurtosis and the family of GLZLM features). It is expected that many of the discretised features will show strong correlation with their non-discretised versions. However, the user must be careful not to remove features that may provide clinical information [42], hence for both radionuclides a separate list of remaining features has been produced that may be applied to local clinical studies on Textural Analysis, where more targeted feature reduction, based on clinical data, can be performed.

The clinical impact of radiomic features in Nuclear Medicine has been highlighted by a number of studies in the literature – mainly in PET, for example showing prognostic value for patient response to treatment [11, 44] or to non-invasively predict tumour metabolic phenotypes [45]. It has been suggested that a lack of reproducible and robust features has led to limited interest in radiomics for SPECT [43], with only a small number of published studies (for example in Parkinson’s Disease [46] and Neuro-Endocrine Tumours [31]). This study aims to promote the identification of robust radiomic features for SPECT imaging through the important initial steps of phantom studies, by adapting techniques recommended for PET imaging to SPECT. It is hoped that by validating the methodology as practicable and relevant to SPECT imaging, it will promote the use of phantom studies in other centres to select robust radiomic features for SPECT imaging and comparison of features across multiple centres will be encouraged to gauge compatibility, with the aim of an increased number of SPECT studies utilising radiomics clinically.

## Conclusion

This study demonstrates that the quality assurance and feature reduction techniques using phantoms proposed in recent Textural Analysis guidelines for Nuclear Medicine can be applied to SPECT for ^177^Lu and ^99 m^Tc radiopharmaceuticals. Features identified as having a strong dependence on volume or high CoV in inhomogeneous lesions may be removed from further analysis, but identification of such features will depend on local acquisition and reconstruction parameters and should be confirmed by other centres.

## Supplementary Information

Supplementary material 1

Supplementary material 2

## Data Availability

The datasets used and analysed during the current study are available from the corresponding author on reasonable request.

## Abbreviations

CoV: Coefficient of variation
EANM: European association of nuclear medicine
LEHR: Low energy high resolution
^177^Lu: Lutetium- 177
MELP: Medium energy low penetration
NEMA: National electrical manufacturers association
PET: Positron emission tomography
SNMMI: Society of nuclear medicine and molecular imaging
SPECT: Single photon emission computed tomography
SUV: Standardised uptake value
^99 m^Tc: Technetium- 99 m
VOI: Volume of interest
