# Supplementary material for: Robustness of textural analysis features in quantitative 99 mTc and 177Lu SPECT-CT phantom acquisitions
Source: EJNMMI Phys. 2025 Apr 17;12:40. doi: 10.1186/s40658-025-00749-0 (PMC12006590; doi:10.1186/s40658-025-00749-0)

Volume dependence of textural analysis parameters for ^{99m}Tc

Uniform phantom of Volume >= 6000 ml

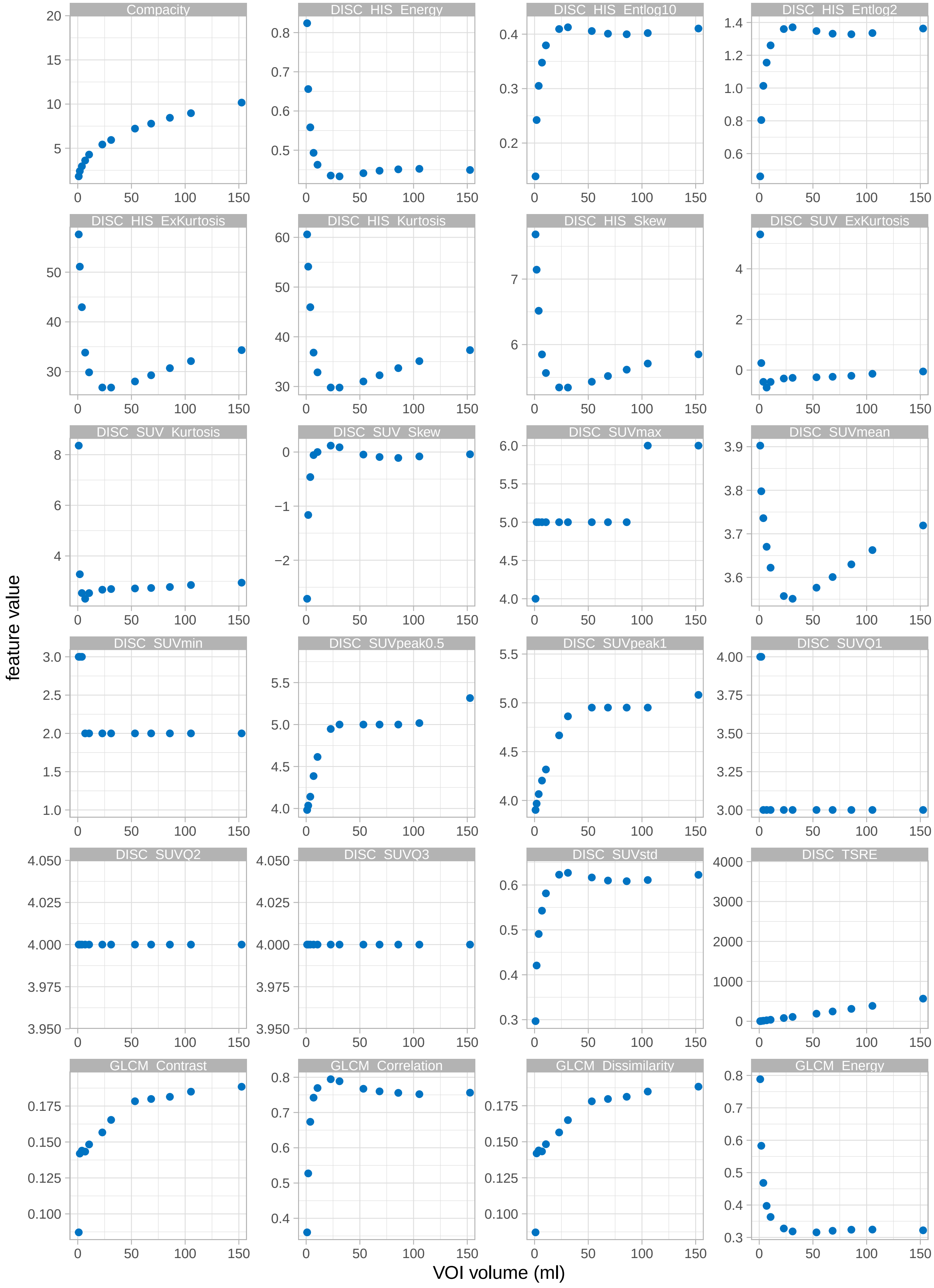

Volume dependence of textural analysis parameters for ^{99m}Tc

Uniform phantom of Volume >= 6000 ml

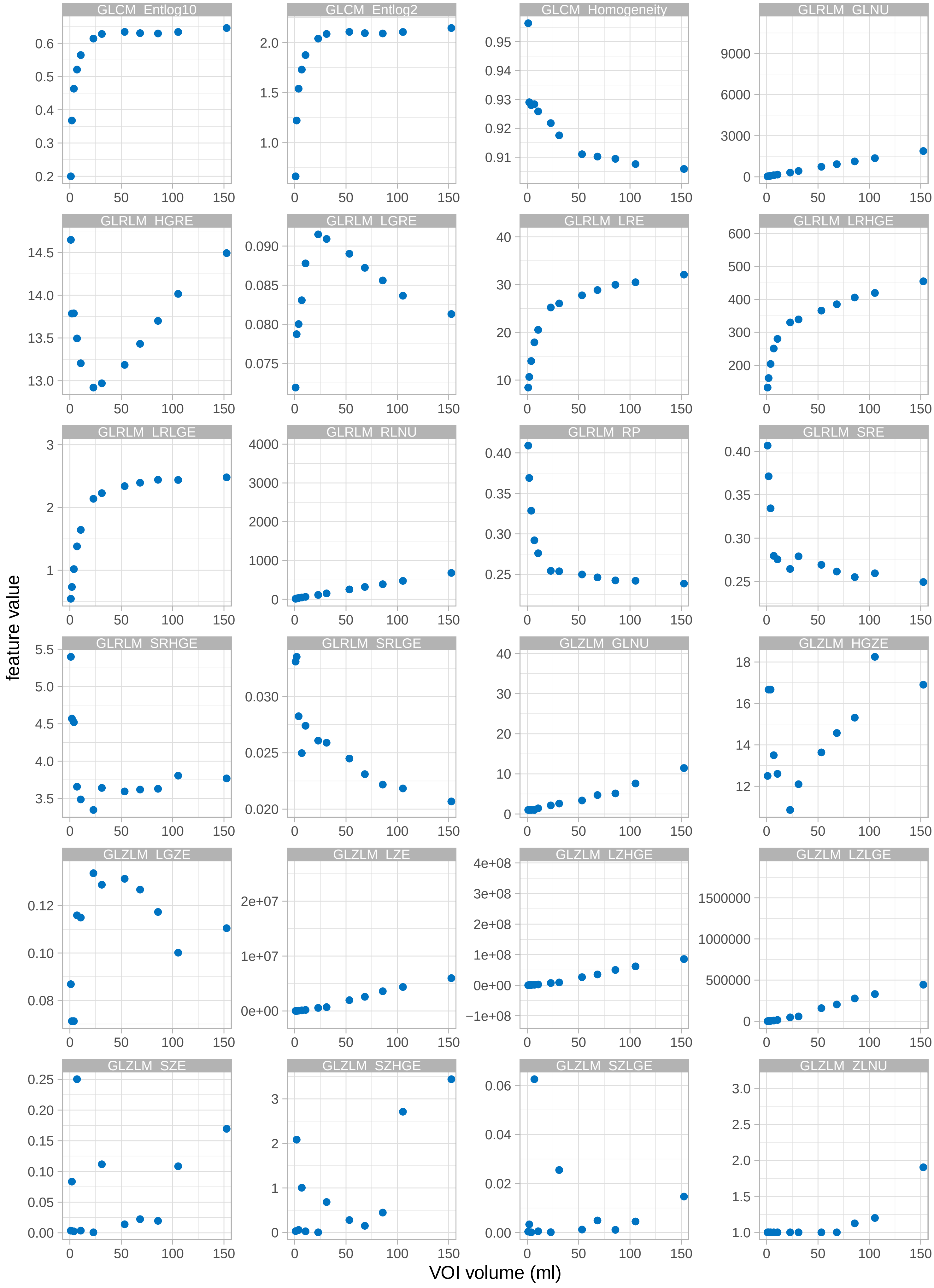

Volume dependence of textural analysis parameters for ^{99m}Tc

Uniform phantom of Volume >= 6000 ml

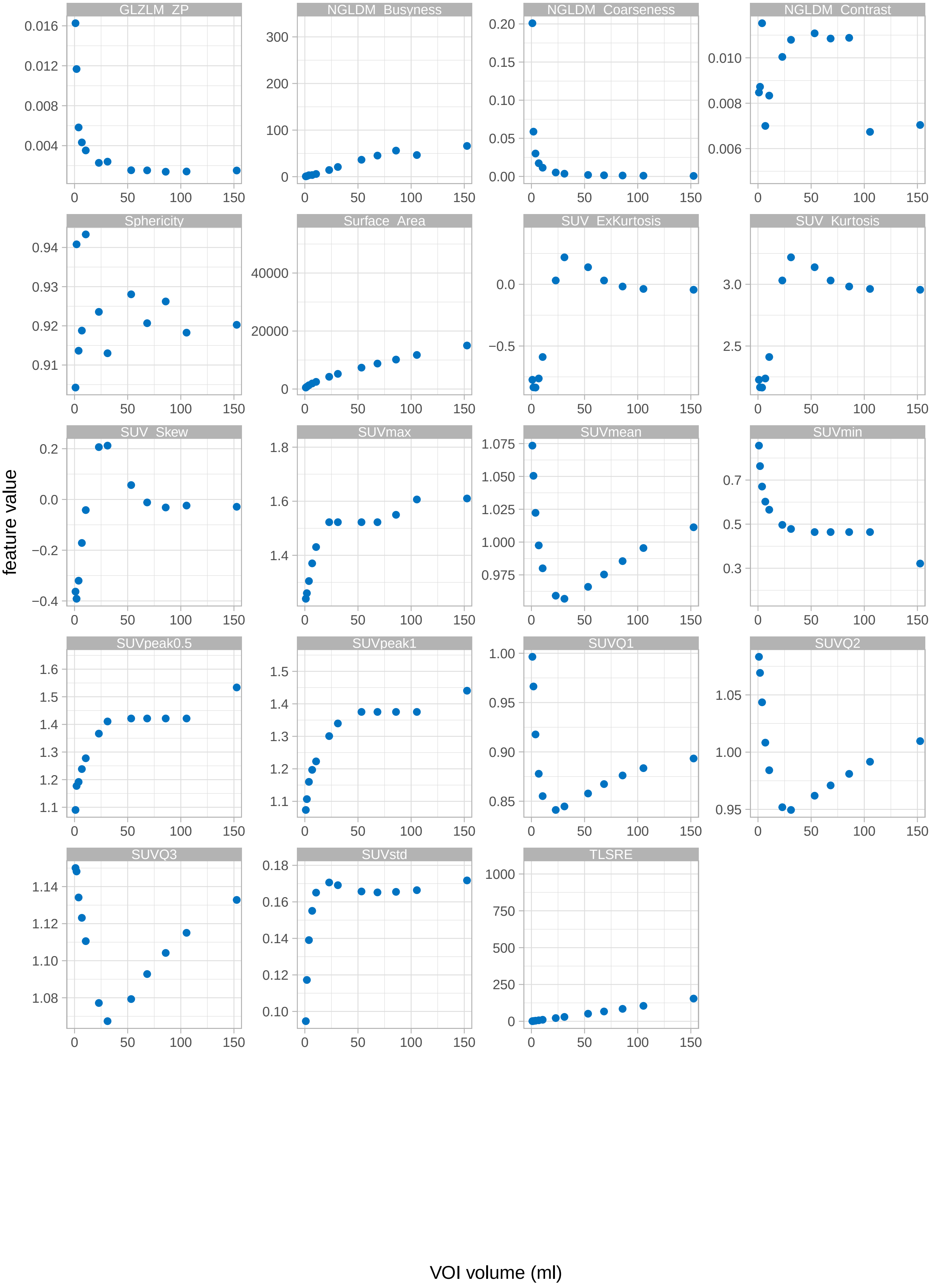

Supplement: Supplementary file 1 — Supplementary material 1 [file 40658_2025_749_MOESM1_ESM.pdf]
