# Supplementary material for: Robustness of textural analysis features in quantitative 99 mTc and 177Lu SPECT-CT phantom acquisitions
Source: EJNMMI Phys. 2025 Apr 17;12:40. doi: 10.1186/s40658-025-00749-0 (PMC12006590; doi:10.1186/s40658-025-00749-0)

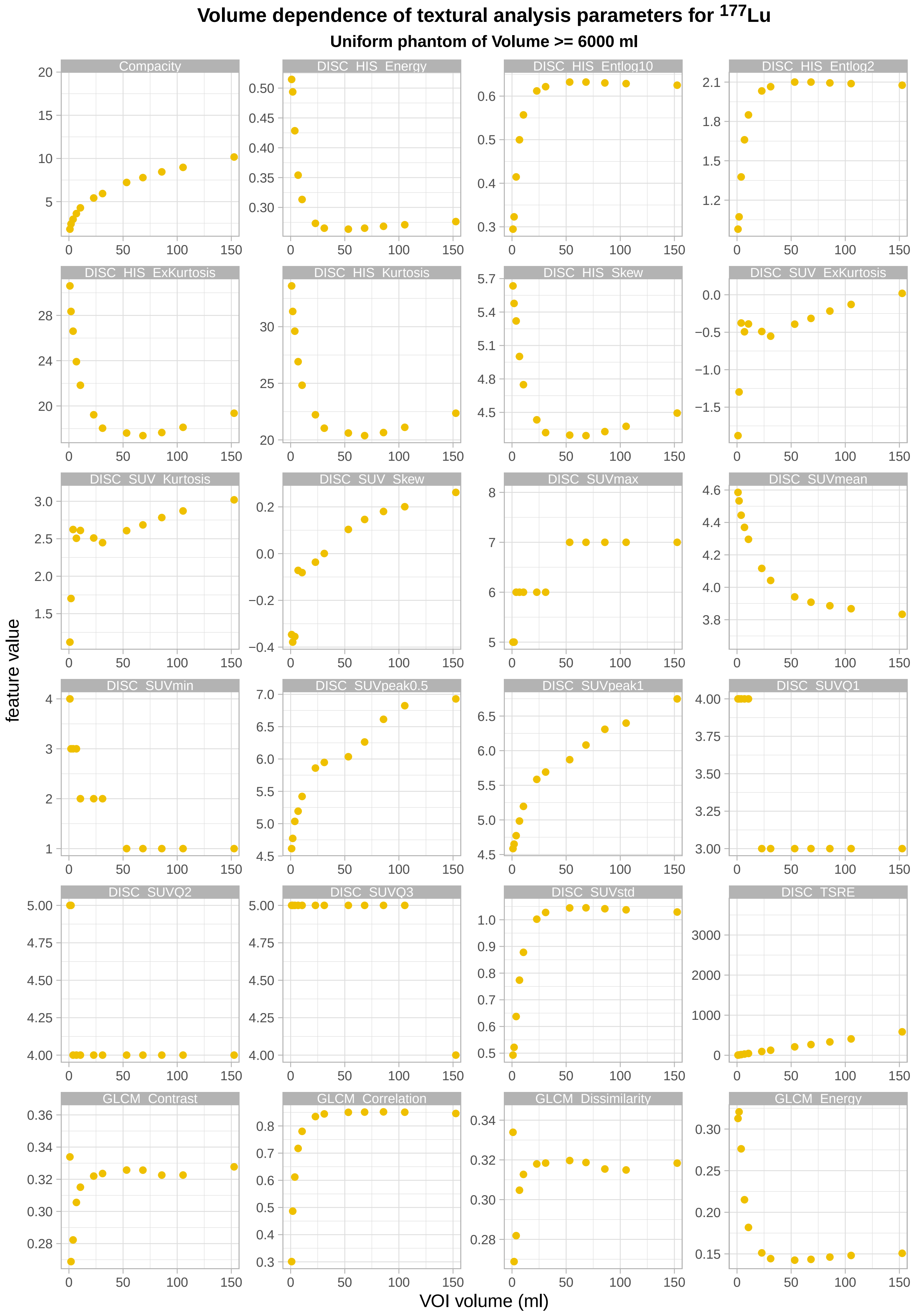

Volume dependence of textural analysis parameters for 177Lu
Uniform phantom of Volume >= 6000 ml
Compacity
DISC_HIS_Energy
DISC_HIS_Entlog10
DISC_HIS_Entlog2
DISC_HIS_ExKurtosis
DISC_HIS_Kurtosis
DISC_HIS_Skew
DISC_SUV_ExKurtosis
DISC_SUV_Kurtosis
DISC_SUV_Skew
DISC_SUVmax
DISC_SUVmean
DISC_SUVmin
DISC_SUVpeak0.5
DISC_SUVpeak1
DISC_SUVQ1
DISC_SUVQ2
DISC_SUVQ3
DISC_SUVstd
DISC_TSRE
GLCM_Contrast
GLCM_Correlation
GLCM_Dissimilarity
GLCM_Energy
feature value
VOI volume (ml)

Volume dependence of textural analysis parameters for ^{177}Lu

Uniform phantom of Volume >= 6000 ml

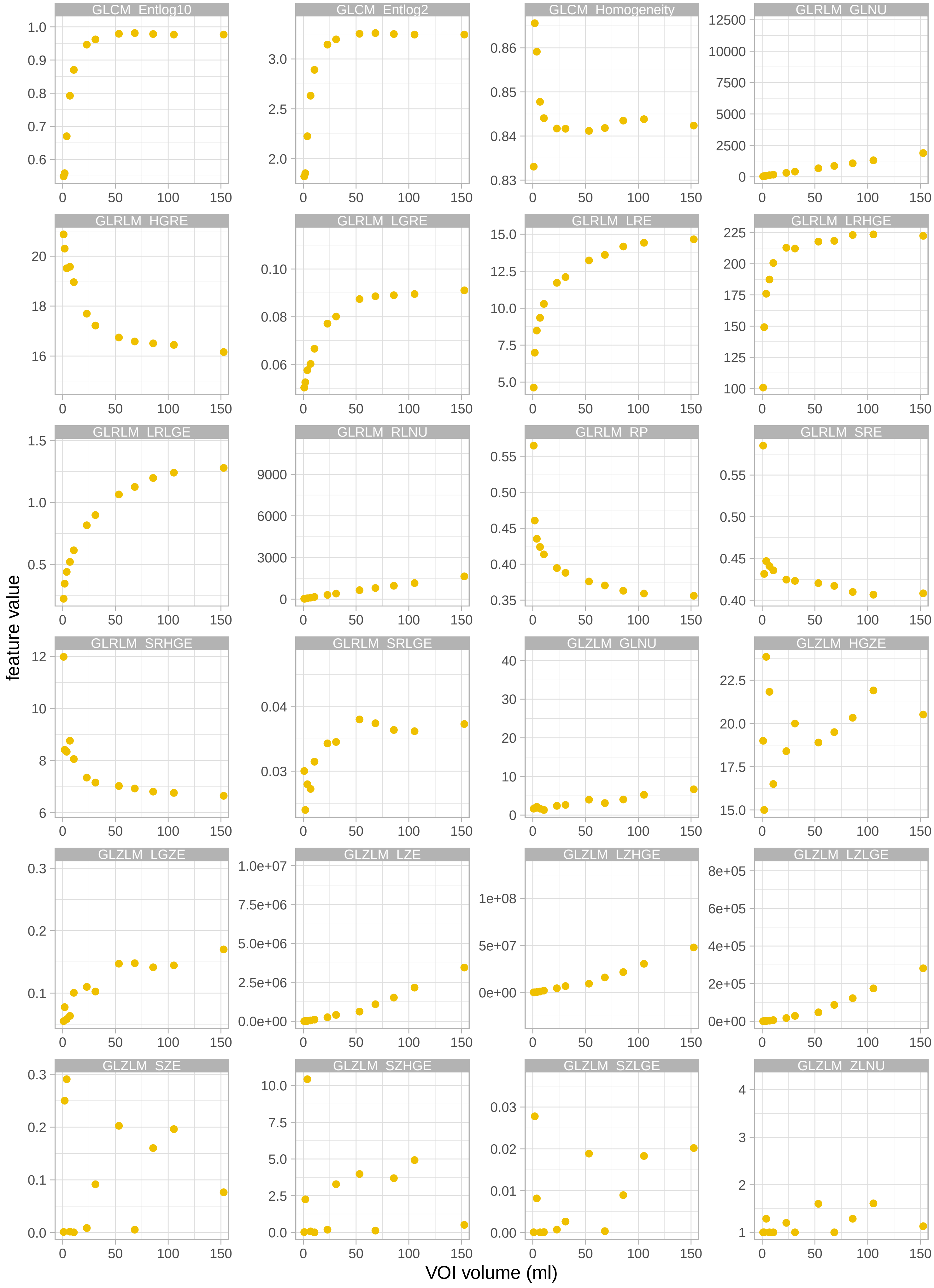

Volume dependence of textural analysis parameters for ^{177}Lu

Uniform phantom of Volume >= 6000 ml

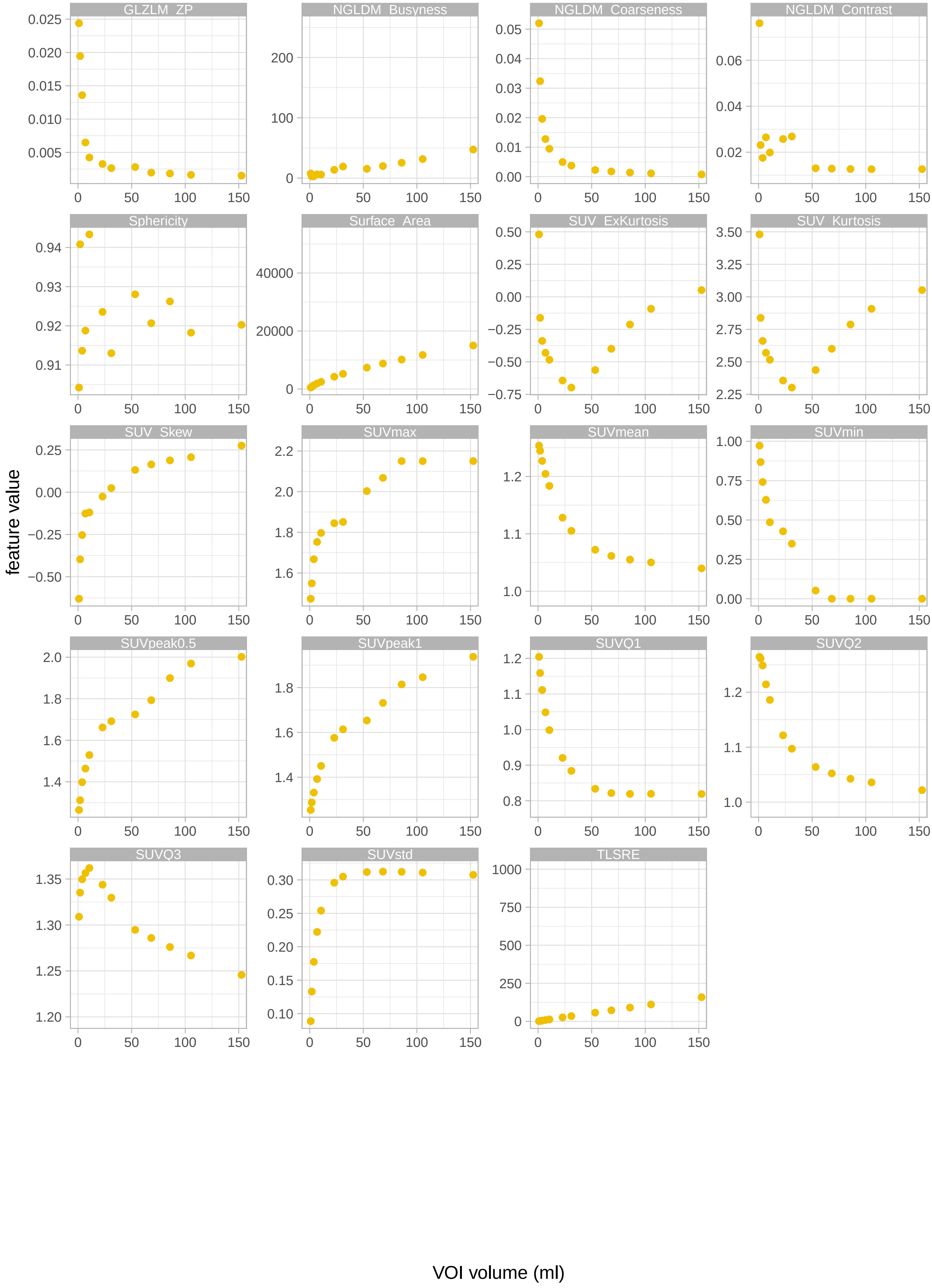

Supplement: Supplementary file 2 — Supplementary material 2 [file 40658_2025_749_MOESM2_ESM.pdf]
